# Experimental determination and ray-tracing simulation of bending losses in melt-spun polymer optical fibres

**DOI:** 10.1038/s41598-020-68568-0

**Published:** 2020-07-17

**Authors:** Birgit Lustermann, B. Maike Quandt, Sebastian Ulrich, Fabrizio Spano, René M. Rossi, Luciano F. Boesel

**Affiliations:** 1 University of Applied Sciences Nordhausen, Weinberghof 4, 99734 Nordhausen, Germany; 2 Empa, Swiss Federal Laboratories for Materials Science and Technology, Laboratory for Biomimetic Membranes and Textiles, Lerchenfeldstr. 5, 9014 St. Gallen, Switzerland; 3 Empa, Swiss Federal Laboratories for Materials Science and Technology, Laboratory of Advanced Fibers, Lerchenfeldstr. 5, 9014 St. Gallen, Switzerland

**Keywords:** Polymers, Theory and computation, Optical materials

## Abstract

The damping properties and specifically the bend losses of polymer optical fibres (POFs) have so far only been documented by experimental work, investigating bending parameters such as bending radius, length, and distance of the bends. Even though damping mechanisms and causes are well-known, no simple, generally valid formula exists. Here, a simulation technique is shown that allows producing an optical model for any bending geometries of melt-spun polymer optical fibres. The developed model takes all relevant loss mechanisms into account, especially regarding the scattering losses at the interface of core and cladding as well as those of the cladding-air interface. The latter is caused by interfacial roughness for which experimental data have been obtained by atomic force microscopy measurements. To show the validity of the simulation, the model is compared to experimental results for several fibres and a variety of geometries. The variance between model and experimental data is low (S < 4.6%). The model not only contributes to improving the understanding of the optical properties of POFs, but it also has direct applicability to the design of photonic textile sensors for medicine, where the fibres are incorporated with small bending radii.

## Introduction

Polymer optical fibres (POFs) are becoming increasingly important in fields such as data transmission (for home, automotive, and industrial use), home illumination, industrial sensors, and healthcare^1–3^. The requirements regarding optical and mechanical properties differ greatly among these diverse applications. While low attenuation losses are extremely important for data transmission applications, they are less relevant for the short transmission paths of biomedical sensors. Here, adequate mechanical properties to withstand sensor fabrication and daily use are paramount.

In recent years, we have used melt-spinning technology to develop POFs with properties similar to traditional monofilament yarn that can be processed with standard textile machinery. They are ductile bi-component fibres made from a cyclo-olefin core and a fluorinated cladding^4,5^. The study and optimization of polymer processing parameters have led to very durable fibres, especially regarding the stability of the core-cladding interface. For their required post-processing, their standout property is the extremely high knot strength with very small bend radii. With these POFs, innovative applications become feasible, such as heart-rate and SpO_2_ sensors^4,6^, or even new phototherapy devices^7^. Melt-spinning has also been used to produce mono-component POFs made from thermoplastic silicone, with applications as < pressure sensors^8^ and breathing sensors^9^. Demonstrating their versatility, several methods, including weaving and embroidery, have been used to produce truly photonic textile medical devices^4,6,7^.

For most of these applications, bending of the POFs is crucial to provide an adequate interaction of the light with the human body. The fibres are bent on purpose to out-couple and in-couple light at defined positions. Hence, knowledge of the bending losses of the polymer optical fibres becomes paramount since the embroidery technique results in extreme bending radii and it is critical to know at which bending radius sufficient outcoupling is reached.

The effect of bending on power loss of POFs has been extensively investigated^10–15^. Eisenstein et al. reviewed works on bending simulations^12^. Numerical simulations, as well as analytical equation-based methods, have been discussed. The latter generally oversimplify the system parameters such as the number of modes, length and geometry of the simulated fibre, leading to inaccurate approximations or to applicability only in single-mode fibres^12^. Moreover, the influence of interfacial scattering, based on measurement data, cannot be found in any of the equations investigated. Numerical simulations such as ray-tracing models or the beam propagation method (BPM) were recommended^12^. Losada and co-workers^13,14^ investigated the effect of bend geometry (ratio of 90° bends to straight, transition sections). Full bends (360°) lead to smaller losses than other geometries, due to the absence of transitional losses^13^, while a single, full turn lead to higher losses than a 90° bend^14^. In general, most of the reported work on bend losses dealt with large bend radii (> 5 mm) and bend angles of 90°–180°^10,11,13–15^ These conditions simulate the typical bending situations found in applications such as data transmission or endoscopy. For applications as sensors, especially textile-based sensors, other conditions need to be met, such as much smaller bending radii (< 1 mm) and the presence of sequential bending loops. In these complex situations, the usable fibre length and, therefore, also the sensor area is ultimately restricted. Investigating the light loss in a controlled setting can then be of help in choosing the most efficient sensor pattern. Recently, Moon et al. used ray-tracing modelling to investigate the effect of curvature on woven POFs^16^. The authors found that an increase in the radius of curvature led to a decrease in light intensity at the end of the fibre tip. This is in disagreement with previous studies, where bending losses were shown to be proportional to the radius of curvature. Moon et al. have, however, used chemically de-cladded fibres, which could have masked the real effect of bending^16^.

In this work, we investigate the effect of extreme radii on bend losses of ductile POFs both experimentally and by using an improved ray-tracing model. The radii simulate those found during textile integration, in particular during the embroidery process. To correctly account for the interfacial and surface scattering, we also present a method (involving topographical measurements as well as simulations) to determine these factors without damaging the fibre or removing the cladding.

## Results

### Experimental determination of bending losses

**Figure 1:**
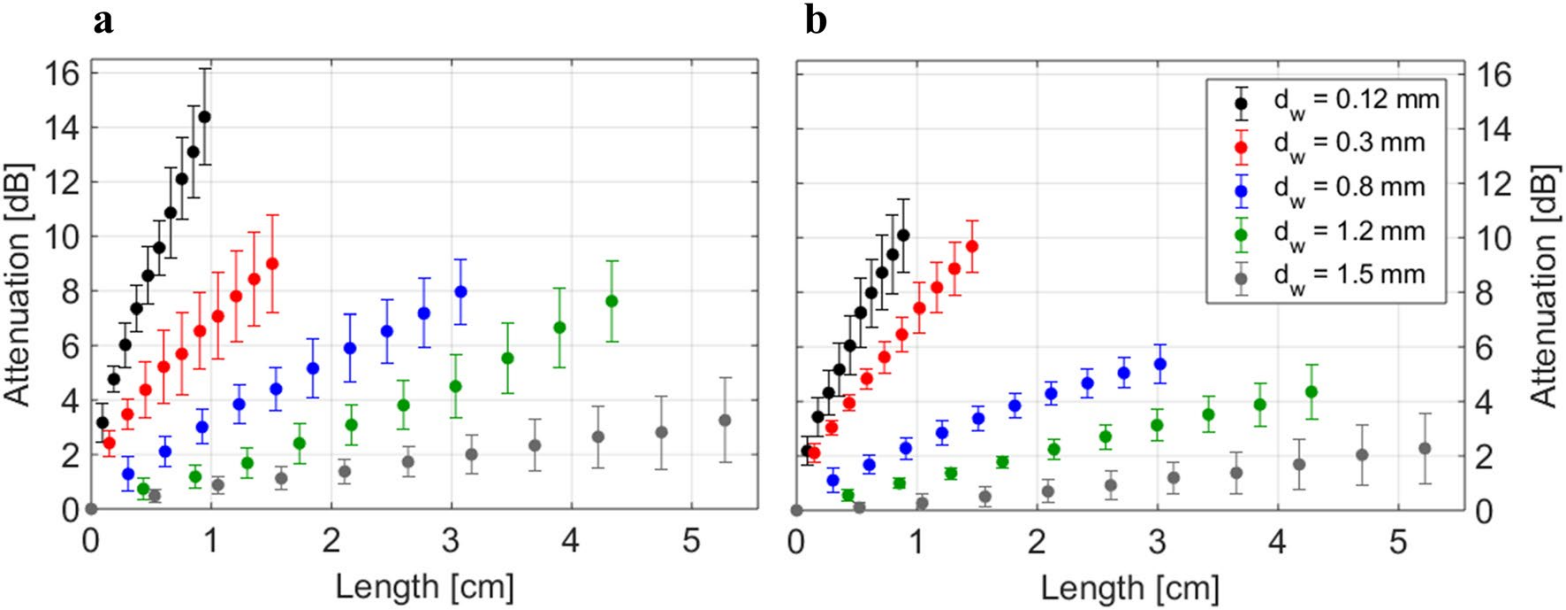
Attenuation *D* of POFs 1143 (**a**) and 1144 (**b**) as a function of fibre length for different diameters of the bending cylinder *d*_w_. Data are shown as mean ± standard deviation (*n* = 10).

Figure 1 shows the attenuation *D* of two melt-spun POFs for several values of bending diameter *d*_w_ as a function of fibre length. The attenuation is defined by

1 $$D = - 10\log_{10} \left( {\frac{{P_{{{\text{out}}}} }}{{P_{{{\text{in}}}} }}} \right)_{{}}$$

where *P*_in_ is the power coupled into the fibre, and *P*_out_ the power leaving the fibre after up to 10 windings (plus the straight segments before and after the bending section, cf. “Methods” section).

The bending losses experimentally increased with the number of bends as expected. The bending radius of the fibre is determined both by the radius of the bending wire and by the radius of the fibre itself, wound around the wire. The generally slight rightwards shift of fibre 1143 (that is, the length in Fig. 1 for any given number of windings is slightly higher for fibre 1143) were, therefore, caused by its slightly larger diameter, cf. Table 1. Except for the wire diameter 0.3 mm, attenuation of 1144 was significantly smaller than for 1143 (see Table S1 and the corresponding discussion).

**Table 1:** Material parameters of the two POFs used in this work. Taken from ^4^, except if stated otherwise. Experimental data displayed as mean ± standard deviation (*n* = 10).

Fiber-ID	*d*_core_ (µm)	*d*_fiber_ (µm)	*n*_core_^a^	*n*_clad_^a^	*α*_core_^a^ [mm^−1^]	*α*_clad_^a^ [mm^−1^]	Damping coefficient (dB cm^−1^)
1143	156 ± 9	179 ± 11	1.53	1.35	2.8∙10^–5^	3.3∙10^–1^	0.16 ± 0.04
1144	141 ± 6*	161 ± 6*	1.53	1.35	2.8∙10^–5^	3.3∙10^–1^	0.14 ± 0.04

*Value significantly different than that for fibre 1143 (*p* < 0.01)^4^.

^a^Data were taken resp. calculated from the datasheets of the polymers^25,26^.

In general, the attenuation shows a non-trivial, non-linear dependence both on fibre length and on bending diameter. In “Modelling of bending losses” section we compare the experimental data to the results of our ray-tracing and scattering model-based simulations, which allow discussing the influence of different attenuation mechanisms in much more detail.

### Determination of the roughness parameters of the cladding-air interface

The roughness of the outer (cladding-air) surface of the fibre is of critical relevance for the simulation of bend losses (in contrast to the simulation of straight fibres) because a substantial amount of modes is out-coupled from the core into the cladding due to bends with a small radius of curvature. Once in the cladding, the modes are reflected and scattered at the cladding-air interface or completely out-coupled from the POF.

**Figure 2:**
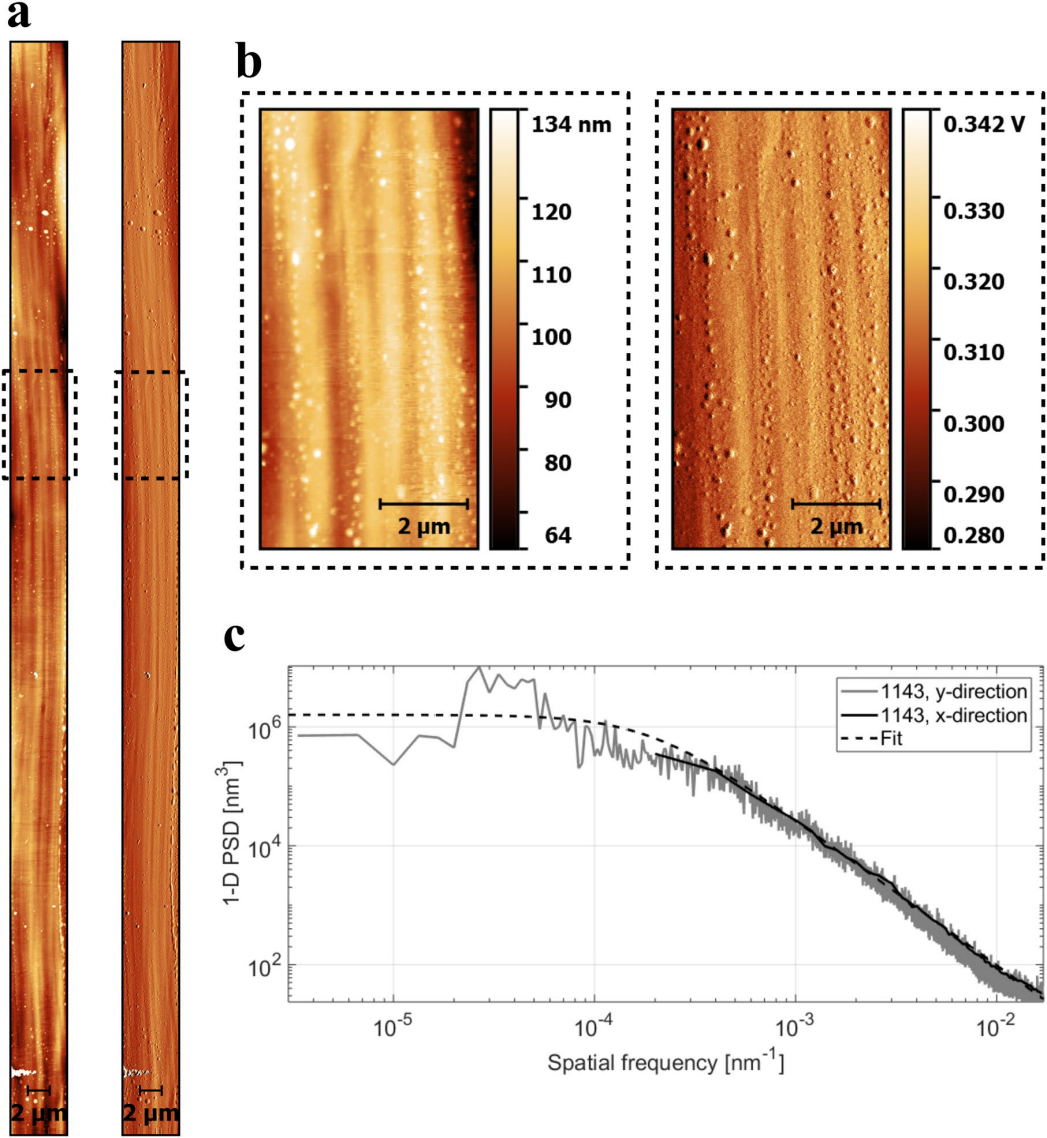
Exemplary atomic force microscopy (AFM) analysis of fibre 1143 at one location. (**a**) Full range AFM image (left: height, right: amplitude). (**b**) Zoom-in of the height and amplitude AFM images in the indicated (5 × 10) µm^2^ section. The depicted height mode AFM images were further treated by a median of differences row alignment. (c) PSD along x- and y-directions for fibre 1143, together with the corresponding ABC-fit curves. The data for fibre 1144 may be found in Figure S1.

As described in “Methods” section, the experimental investigation of the cladding-air interface was performed by atomic force microscopy. Figure 2a, b show full range as well as zoomed-in areas of the fibre 1143 surface. The data for fibre 1144 may be found in Figure S1. The inhomogeneities were roughly aligned along the main fibre axis and consisted of spherical, nano-sized protrusions that clearly affect the height profile. The profile was also affected by the surface structuring arising from the melt extrusion process, clearly visible in Fig. 2a, b as striations parallel to the fibre axis.

Based on the obtained height profiles in x- and y-directions for each scan, the power spectral density (PSD) was calculated (cf. Eq. (8), “Methods” section). To determine the ABC fit, and from them, the root mean square (rms) roughness of the surface, these PSD were averaged and subsequently fit, see Figs. 2c and S1c. One recognizes the typical flattening of the averaged PSD towards low spatial frequencies caused by the finiteness of the sample. Note that the y-PSD reaches much lower frequencies (see the grey curve in Fig. 2c). We performed the AFM measurement over a continuous length of 100 μm in the direction of the fibre axis which allowed the assessment down to very low frequencies. Measurement perpendicular to the fibre axis was naturally more limited by the fibre diameter (< 200 μm) and curvature. The difference between x- and y-PSD concerning the amplitude of fluctuation was caused by the different number of averaged individual line profiles: There were 5,210 PSD calculations along the x-direction, but 256 PSD calculations along the y-direction. The fluctuation of the PSD towards lower spatial frequencies was much stronger than at higher frequencies. This statistical error was caused by the limited scan length and the increase of surface impurities at larger scales^17–19^.

The ABC fits of both fibres gave the following stray light model parameters and roughness values:

2 $$\begin{gathered} {\text{Fiber}}\,{1143}:A = {3}.{19} \cdot {1}0^{{6}} ,B = {5}.{28} \cdot {1}0^{{3}} ,C = {2}.{45} \to S_{q} = {27}.{2}\,{\text{nm}}\,{\text{with}}\,{\text{slope}}\,sl = 0.0{24}. \hfill \\ {\text{Fiber}}\,{1144}:A = {7}.{35} \cdot {1}0^{{5}} ,B = {2}.{39} \cdot {1}0^{{3}} ,C = {2}.{48} \to S_{q} = {19}.{3}\,{\text{nm}}\,{\text{with}}\,{\text{slope}}\,sl = 0.0{37} \hfill \\ \end{gathered}$$

### Determination of the roughness parameters of the core-cladding interface

**Figure 3:**
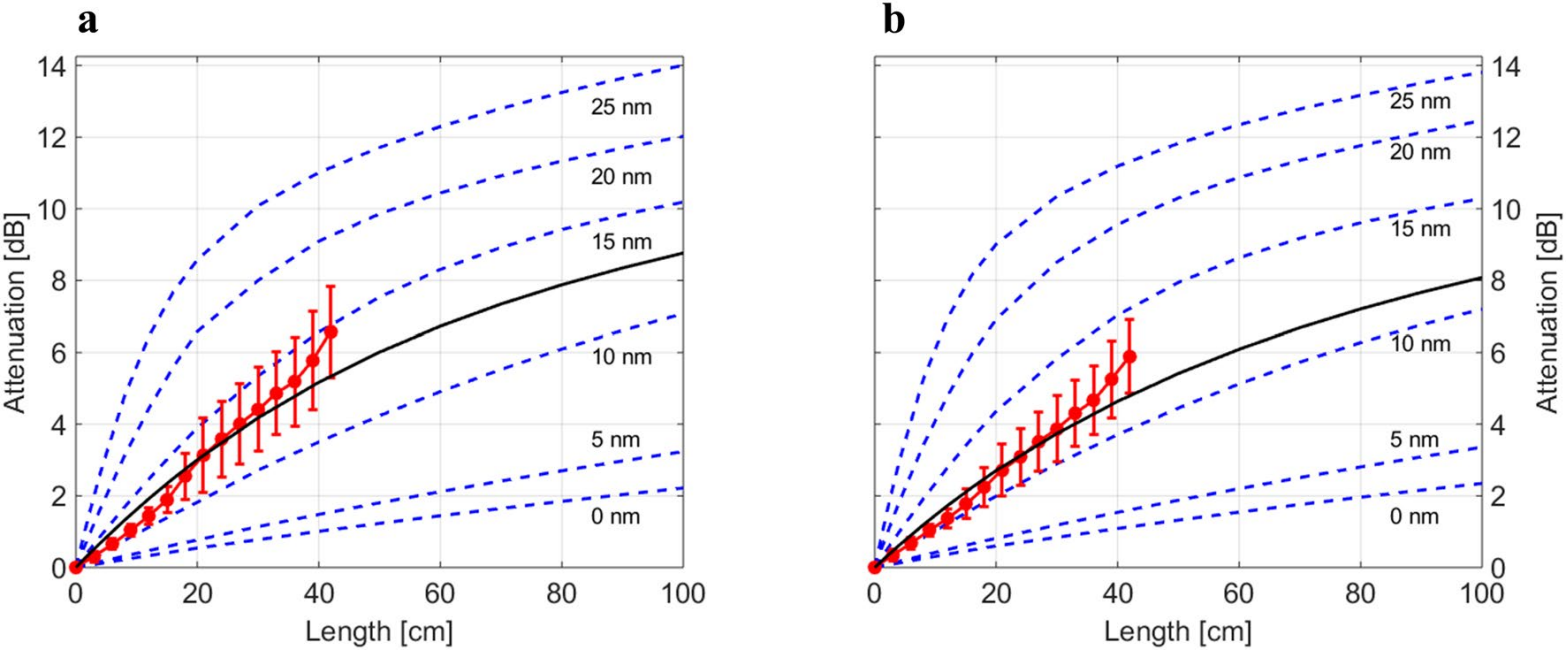
Measured data for the straight fibres (red circles: mean with standard deviation, *n* = 10) compared to simulation results for various values of the rms roughness of the core-cladding interface *S*_*q*_ = [0 … 25] nm (blue dashed lines). The solid black line represents the best interpolation between the simulation for *S*_q_ = 10 nm and *S*_*q*_ = 15 nm based on a least-square fit for the respective fibre: 1143 (**a**), 1144 (**b**).

As mentioned in “Methods” section, the core-cladding interface is not accessible to direct measurements. Hence, we chose to determine the roughness parameters from experimental data for the damping behaviour of the straight fibres (Fig. 3).

Considering first the propagation of rays without modelling the core-interface roughness, i.e. taking into account only the refractive indices and the absorption coefficients of core and cladding, the lower curves in Fig. 3, corresponding to *S*_*q*_ = 0, were obtained. The obtained losses clearly underestimate the measured attenuation values. Therefore, a stray light model was assigned to the core-cladding interface which allowed the simulation of fictitious roughnesses of the interface (numerical investigations have shown that for sufficiently long straight fibres the outer, i.e. the cladding-air, the interface does not influence the guiding of light and can be neglected)^20^. For this purpose, the ABC parameters described earlier were calculated to obtain values for the rms roughness *S*_*q*_ in the range 5–25 nm, which are typical for polymer optical fibres^21^. From a number of material investigations, it is known, that the parameter *C* entering Eqs. (9) and (11) typically is ≈ 2 and that, for the materials chosen here, the slope parameter *sl* is of the order of 0.02–0.04^20,22,23^. The measurement of the cladding-air surface described above confirms these values, see Eq. (2). Moreover, in order to determine the effect of changes in *C* and *sl*, we simulated the damping behaviour for values of *C* between 1.8 and 2.5, keeping constant *S*_*q*_ and *sl*, as well as for values of *sl* between 0.018 and 0.04 keeping constant *S*_*q*_ and *C*. These simulations resulted in a systematic error of less than 0.3% caused by the uncertainty of parameter *C* and 0.1% caused by the uncertainty of parameter *sl*. In the following we, therefore, assume for the calculation of *S*_*q*_ a slope of *sl* = 0.02 and a value of *C* = 2.

Comparing the measured data to the simulation results for different values of *S*_*q*_, the rms roughness of the real core-cladding interface of the investigated fibres was found to lie between 10 and 15 nm, see Fig. 3. In order to derive a more precise value, an interpolation between the simulation results for these two roughnesses was performed and the best least-square fit to the experimental points was determined. The resulting values for *S*_*q*_ are summarised in Table 2.

**Table 2:** Values of interface roughness, used for simulation of bent fibres.

Fiber-ID	Core-cladding interface *S*_*q*_ (nm)	Cladding-air interface *S*_*q*_ (nm)
1143	13.2	27.2
1144	11.8	19.3

### Modelling of bending losses

**Figure 4:**
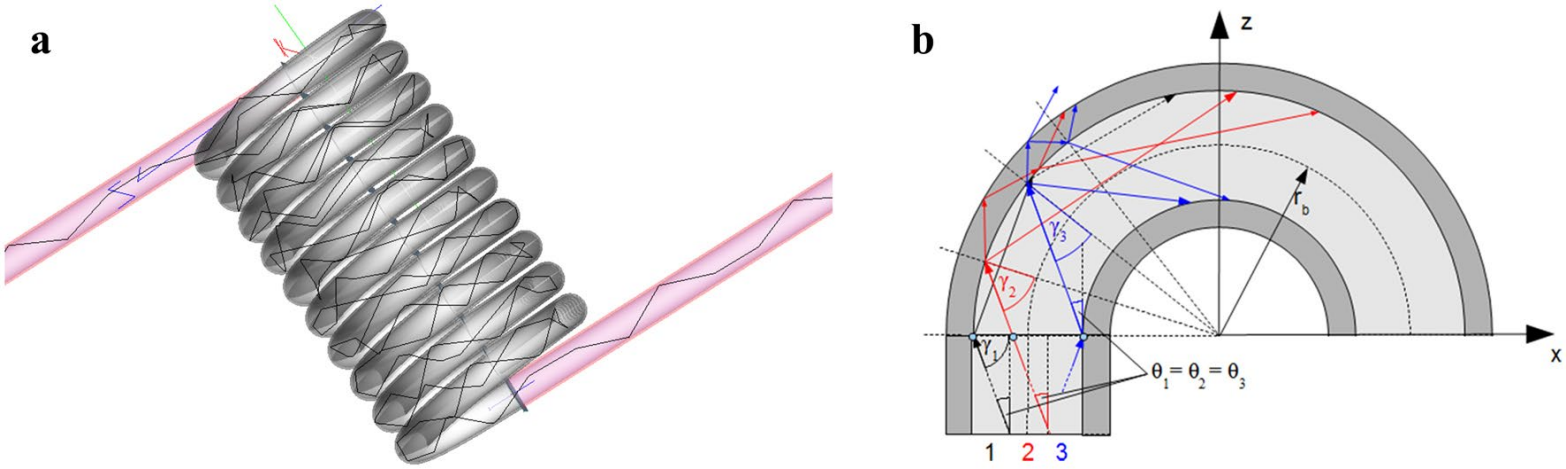
(**a**) Ray-tracing model with 10 bends (not to scale). (**b**) Ray paths within a bent fibre for several entry points with the same polar angle θ.

In a straight fibre, attenuation essentially depends on the interfacial structure between core and cladding. If, on the other hand, an optical fibre is strongly bent, the scattering on the outer surface of the cladding also plays a decisive role in the fibre losses. Indeed, any bending of fibres leads to modified reflection conditions. Especially rays, which are guided in a straight fibre, could impinge onto the core-cladding interface at an angle violating the total reflection condition. The light will be partially coupled into the cladding or leave the fibre (“Fresnel losses”). Whether a ray will be guided under the acceptance angle of total reflection in the core (black ray in Fig. 4b), coupled and guided in the cladding (red ray in Fig. 4b) or will be outcoupled (blue ray in Fig. 4b) depends, among other parameters, on the position of the entry point into the bend and the relation between bending and core radius, cf. the different start positions of the rays in Fig. 4b. Additionally, the guiding through the cladding and, therefore, the roughness of the cladding-air-interface, is more important in bent fibres compared to straight ones. The bends allow a ray to reach the interface at more favourable angles for guiding within the cladding.

The relevant damping mechanisms for bent fibres are, therefore:

- The initial reduction of the guided beam angle due to outcoupling (bending losses caused by the transgression of the angle of total reflection at the cladding-air interface, “Fresnel losses”),
- The attenuation inside the cladding, in particular scattering at the cladding-air interface and absorption in the cladding,
- The attenuation inside the core, caused by scattering at the core-cladding interface and the (negligible small) absorption of the core material.

The geometrical parameters influencing the conditions of total reflection and therefore the bending losses are the bending radius and the pitch (distance between the centre of adjacent windings). The bending radius was determined by the diameter of the bending wire *d*_w_ and the specific fibre geometry. For both fibres the simulations were performed assuming a pitch distance of 0.18 mm which is the diameter of fibre 1143 (plus 1 µm required by the simulation software in order to have uniquely defined interface boundaries). During the metrological tests the fibres were wound as tightly as possible without touching each other. The fibre distance was not greater than approx. 1 mm, but was not exactly determined. Investigations on the influence of the pitch distance on the transmitted power of the bent fibre showed that values up to 1 mm lead to only slight changes (see Fig. S3 for an example of 3 bending diameters for fibre 1143) and that the pitch is less relevant for intermediate and high *d*_*w*_. The simulation results showed in particular that larger distances (pitch > 1 mm) lead to worse simulation results. Therefore, the above-mentioned pitch value of 0.18 mm was uniformly used for all simulations. For the stray light model of the cladding-air interface, the parameters *A*, *B*, and *C* are given by Eq. (2); for the model describing the core-cladding interface they were calculated from the determined rms roughness *S*_*q*_ (see Table 2) using Eqs. (10) and (11) assuming a slope value of 0.02 and *C* = 2 (cf. the explanation in “Determination of the roughness parameters of the core-cladding interface” section).

**Figure 5:**
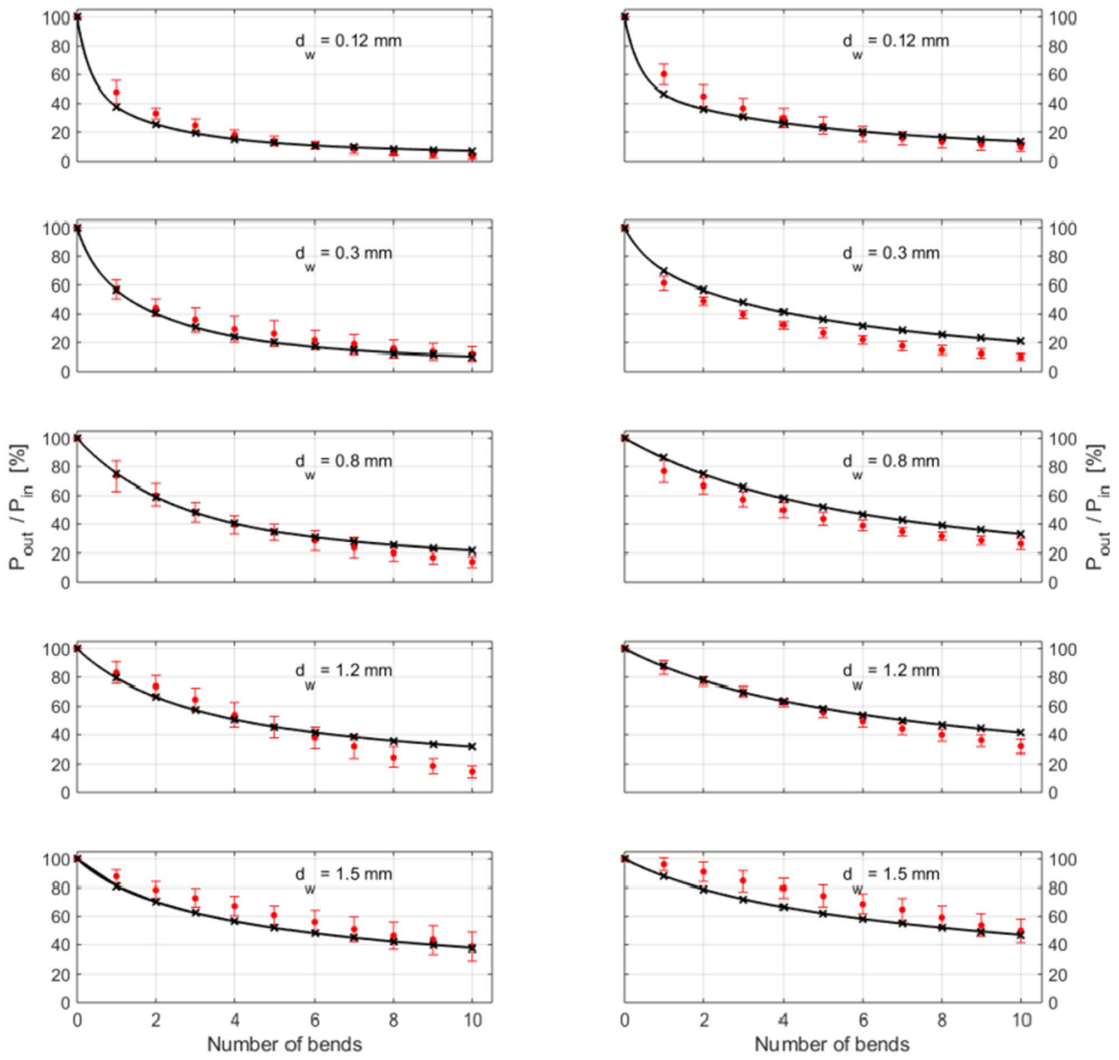
Comparison of measured and simulated data for all bending radii, for both fibres (left: fibre 1143; right: fibre 1144). Circles with error bars: measured data (*n* = 10); crosses: ray-tracing results; solid lines: fitting according to Eq. (4).

Applying the derived stray light models, the bending behaviour of both fibres could be simulated and compared to the experimental data, see Fig. 5. The overall quality of the simulation is very good, especially given the experimental difficulties mentioned earlier.

The power ratio P_out_ / P_in_ at the end of the fibre is connected to the attenuation *D* used above by inverting Eq. (1),

3 $$\frac{{P_{{{\text{out}}}} }}{{P_{{{\text{in}}}} }} = 10^{{ - \frac{D}{10}}}$$

Analysis of the outcoming power (instead of the attenuation) has the advantage that each individual loss mechanism can be expected to lead to an exponential decrease of power with an increasing number of bends (and, consequently, length) of the fibre.

**Figure 6:**
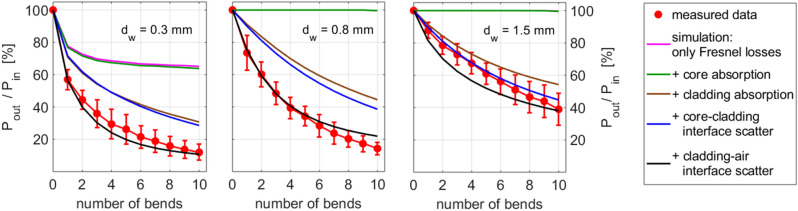
Comparison of the experimental bend losses (red circles: mean with s.d., *n* = 10; red line only to guide the eye) of fibre 1143 for three wire diameters *d*_w_ with models incorporating different loss mechanisms. Pink line: model incorporating only Fresnel losses; green line: model incorporating Fresnel losses and core absorption; brown line: model incorporating Fresnel losses and core and cladding absorption; blue line: model incorporating all previous mechanisms plus the scattering at the core-cladding interface; black line: complete model incorporating all 5 loss mechanisms.

Regarding the individual loss mechanisms listed above, Fig. 6 shows the contribution of each of them to the outcoming power of the bent fibre for several values of the wire diameter *d*_w_. An, at least qualitative, agreement to the experimental data was only achieved when all loss mechanisms were taken into account. Initially, all losses based on any kind of absorption and scattering were neglected (pink line). In Fig. 6 only the small value of *d*_w_ (0.3 mm) lead to losses caused by exceeding of the angle of total reflection (Fresnel losses), whereas with larger values of *d*_w_ (0.8 mm, 1.5 mm) the Fresnel losses were nearly zero (the line is not visible under the green line in both cases). The implementation of the core absorption (green line) in the ray tracing model had no relevant contribution to bending losses for any investigated bending radii. In contrast, the introduction of the cladding absorption (brown line) lead to much more losses. However, the observed behaviour can only be reproduced when considering the interface scattering. First, the additional core-cladding interface scatter was considered, and an increasing influence of this loss mechanism with increasing bending diameter (blue line) was observed. For small values of *d*_w_ (0.3 mm, 0.8 mm) there was still a big difference between measured und simulated results, which could be matched only when the scattering of the cladding-air interface was also taken into account (black line). Moreover, in accordance with the explanation given at the beginning of “Determination of the roughness parameters of the cladding-air interface” section, with increasing bending radius, the role of cladding-air scattering decreases, leading to a convergence between the black and the blue curves in Fig. 6. A more detailed discussion will be given in the next section.

In order to analyse the behaviour of the different loss contributions quantitatively, a fit formula for the simulation results was developed. Since each of the loss mechanisms lead to an approximately exponential decrease in the power of the guided light, we assumed a fit of *P*_out_ / *P*_in_ as a sum of three exponentials, each corresponding to these three different damping mechanisms,

4 $$\frac{{P_{{{\text{out}}}} }}{{P_{{{\text{in}}}} }}\left( {d_{{\text{w}}} ,{ }N} \right) = P_{1} \left( {d_{{\text{w}}} } \right) \cdot \,{\text{ e}}^{{ - Np_{1} \left( {d_{{\text{w}}} } \right)}} + P_{2} \left( {d_{{\text{w}}} } \right) \cdot \,{\text{e}}^{{ - Np_{2} \left( {d_{{\text{w}}} } \right)}} + P_{3} \left( {d_{{\text{w}}} } \right) \cdot \,{\text{e}}^{{ - Np_{3} \left( {d_{{\text{w}}} } \right)}}$$

where *N* indicates the numbers of bends. The values for each of the six parameters *P*_*i*_ and *p*_*i*_ (*i* = 1, 2, 3), for each fibre and as functions of the bending diameter *d*_w_, are presented in Tables S2 and S3 and visualized in Figure S2. Since for *N* = 0 (i.e. no bends) *P*_out_*/P*_in_ equals 1, *P*_*1*_ + *P*_*2*_ + *P*_*3*_ has to be = 1 and, therefore, for each *d*_w_ five parameters have to be fit. Performing a least-square fit between Eq. (4) and the simulation results for bending numbers *N* = 1, 2,…,10 and the specific bending geometries investigated in the present paper gives an excellent agreement with deviations well within the simulation accuracy of 1% (cf. Section “Methods”), see Fig. 5. The fit also correctly represents the experimental data, as inferred by the small values of *S* (the standard error of the regression) in Tables S2 and S3: *S* < 2.4% for fibre 1143 and *S* < 4.6% for fibre 1144.

## Discussion

In general, the two fibres investigated did not show strong differences in performance and had much lower bending losses than conventional POFs^13^. For a more extensive study on melt-spun fibres, it would be interesting to investigate whether the bending behaviour can be linked to a production parameter. E.g., Durana et al. showed a dependence of bend losses on the cladding thickness^15^. Here, we only changed the draw ratio (2 for fibre 1144 and 1 for 1143) and hot-drawing temperature (90 °C and 40 °C, respectively). These parameters did not have such a strong effect on bending behavior as on the mechanical properties^4^.

In accordance with the method of measurement, our model incorporates three segments: first, a straight segment (6 cm), second, a segment with 10 windings (one detector after every winding) and, third, a straight segment again, see Fig. 4a. The material parameters used for the simulation are the refractive indices and the absorption coefficients of core and cladding (see Table 1). The number of rays during the simulation must be large enough to reduce the simulation uncertainty so that it is much smaller than the error of the measurements. In our case, in each simulation we traced 10^5^ rays resulting in an uncertainty of the predicted power loss of <  ± 1%. The uncertainty of the model is, therefore, well below that of the experimental data (see Fig. 1). The deviations between experimental and modelled data seen in Fig. 5 are therefore most probably the result of experimental errors such as variations in the pitch distance among different bends, inhomogeneities in the fibre itself, or errors arising from the in-coupling of light (both direction of in-coupling as well as the imperfect surface of the fibre). These three errors can also be assumed in the measurement of the straight fibre which in turn influences the determination of the core-cladding interface roughness. This roughness value adds further uncertainty to the simulation of the bends. In addition, the simulation did not take into account the effect of closely opposing windings leading to possible tunnellings of light rays from one winding to the next one.

The exponents of the three loss components differ one from each other almost by a factor of 10 (Figure S2 and Tables S2 and S3), especially for small bending radii, and decline with increasing *d*_w_. This corresponds to the fact, that for *d*_w_ → ∞ the behaviour of the straight fibre is approached, for which losses are due only to core-cladding scattering, cladding absorption and (the negligible small) core absorption. On the other hand, the *d*_w_-dependences of the amplitudes are qualitatively different one from each other: Whereas amplitude *P*_*1*_ goes down with increasing bending radius, *P*_*2*_ has a maximum for intermediate *d*_w_ and *P*_*3*_ increases roughly linearly with increasing *d*_w_.

For very small *d*_w_ (< ≈ *d*_core_), a large part of the power is coupled out from the fibre due to refraction of rays with large angles (relative to the fibre axis) from the core into the cladding and afterwards from there into the air (“Fresnel losses”). Since the number of light rays fulfilling the condition of total reflection increases with decreasing core radius of the bent fibre^20^, the relevance of Fresnel losses decreases with decreasing core radius. For fibre 1144, having a smaller core radius compared to 1143, the coefficient *P*_*1*_ is slightly smaller, confirming this hypothesis.

For larger *d*_w_ (> 0.8 mm), there remain two essential sources of loss: the attenuation in the cladding, in particular the material absorption in the cladding, and the scattering at the cladding-air surface, and the attenuation in the core including core-cladding scattering and the (negligible small) core material absorption.

The behaviour of the amplitude *P*_*2*_ and of the exponent *p*_*2*_ (which is approximate of an order of magnitude larger than the third one, *p*_*3*_) suggest that the contribution to the total loss corresponds to cladding effects, namely the scattering at the cladding-air interface and the material absorption in the cladding. Indeed, *P*_*2*_ reaches its maximum for the value of *d*_w_ at which the Fresnel losses vanish (0.8 mm), and decreases afterwards. At the same time, the relevance of the third damping contribution, *P*_*3*_, increases with increasing bending radius. This suggests that the third contribution to Eq. (4) corresponds to core effects, specifically to the core-cladding scattering and to the (very small) material absorption in the core, which provides the dominant contribution to the attenuation of straight fibres.

Another argument for this interpretation of the third contribution stems from the fact that, for small bending radii, for fibre 1144 both the coefficient *P*_*3*_ and the exponent, *p*_*3*_, are larger (as compared to 1143). This can be explained by the smaller core diameter of fibre 1144, leading, for a given length of the fibres, to a larger number of reflections at the core-cladding interface and, therefore, both to more rapid damping and to larger importance of core effects.

## Conclusion

In conclusion, we developed a method to estimate the bending losses of POFs of arbitrary geometry for any bending radius with remarkable accuracy by using a ray-tracing approach. Our model clearly shows that all relevant loss mechanisms of bent embroidered fibres could be considered. In particular, it includes the scattering at both interfaces, which is an important factor for the total bend losses that has not been investigated in detail before. An essential prerequisite for this was the experimental investigation of the damping behaviour of the straight fibre, from which the roughness parameters of the core-cladding interface could be derived. Having the roughness parameters, an integrated surface scatter model could be built and included into the simulation, leading to a good agreement between simulated and measured data. Remaining differences between experimental values and simulation results arose from the inhomogeneities of the fibres, the uncertainties of the values of attenuation coefficients of the cladding and the interface roughness, as well as the deviation between experimental and simulation parameters (i.e. pitch angle).

This model allows the prediction of the optical response of our fibres when integrated into textiles by simply measuring a set of basic properties (those shown in Table 1) as well as the cladding-air surface roughness. Of special importance is the applicability of the method to radii typically found during the preparation of embroidered photonic textile sensors, that is, lower than 0.5 mm.

Further work shall be done on investigating these models over other relevant regions of the spectrum, e.g., the blue region or the near IR (from 600 to 1,000 nm). For the future, we also plan to examine the influence of the assumed vertical and lateral structures of the core-cladding interface and the material absorption coefficients to achieve a good agreement over all measured data sets. The developed integrated ray-tracing model will help to guide the further development of photonic textile sensors incorporating POFs bent with extreme radii.

## Methods

### Statistical data analysis

was performed with the “R" program^24^ and the “R-commander" package. Shapiro–Wilk tests were used to test for the normality of the samples; afterwards, differences in the central values of two samples were tested by using the Student t-test. One-way analysis of variance (ANOVA) was used to test for differences in regression slopes. The analysis results are noted at the respective images or tables.

### Bending experiments

The production and characterization of the used POFs have been described previously^4^. The main properties of the fibres relevant to this work are displayed in Table 1. Given their ductility and knot strength, the fibres can be wound around small-diameter wires. Both used fibres show high homogeneity in their cross-sections and, correspondingly, also in their mechanical properties^4^.

Several different bending radii were investigated, defined by wires (0.12–1.5 mm diameter) around which the POF was wound with 10 full rotations (see Fig. 4a). For this purpose the POFs were attached to a mode mixer via F-SMA connectors (Thorlabs, Newton, NJ, USA). For illumination, a medical laser diode was used at 100 mW with a wavelength of 652 nm (Applied Optronics, South Plainfield, NJ, USA). The fibres were then kept in place by double-stick tape (at a distance of 2 cm both in front and behind the wire). The total length of the fibre used in the experiments was 70 cm: there was an initial section of straight fibre (6 cm) before wounding around the wires; the bended section was variable, depending on wire diameter and number of bends, and amounted to a maximum of 5.5 cm; last, there was a long section of straight fibre. The fibre end was attached to the integrating sphere (UM-150, Gigahertz-Optik GmbH, Türkenfeld, Germany) at a 2 cm offset from the fibre end. The integrating sphere was connected to a photomultiplier tube (PRC Krochmann, Berlin, Germany). Experiments were replicated 10 times with different fibre sections for each bending radius and fibre type.

The attenuation was measured by cutback technique as described elsewhere^5^, using the apparatuses as explained in the previous paragraph. The output power *P(L)*, in dependence of the fibre length *L*, was determined by subsequently shortening the fibre, while keeping the input coupling to the fibre constant.

For the simulation, further parameters included refractive indices of core and cladding (*n*_core_*, n*_clad_), core diameter (*d*_core_), fibre diameter (*d*_fiber_), absorption of the materials at the used wavelength (*α*_core_, *α*_clad_) and length of the used fibre section (see Table 1). For the simulation of bending losses, the same geometry was used as described above for the bending experiments.

### Measurements of the cladding-air surface roughness

An important source of losses in POFs is the interface roughness of the fibre, both between core and cladding and between cladding and air. The measurement of the height profile of the interfaces constitutes the basis for a scatter model which describes the effects of reflection and refraction at rough interfaces. For the outer interface of the fibre these roughness measurements can be obtained by atomic force microscopy (AFM) experiments with sufficiently high resolution (in the nm-range) for relatively large areas. The AFM analysis was performed by means of a scanning probe microscope FlexAFM V5 (Nanosurf AG, Liestal, Switzerland) equipped with a C3000 controller and the associated software. The measurements were accomplished in tapping mode using a pyramidal silicon tip with a resonance frequency of 190 kHz and a spring constant of 48 N m^−1^ (BudgetSensors, Tap190Al-G). For one image, 5,120 lines of 5 µm length in direction of the x-axis (perpendicular to the fibre axis) with a scanning time of 1.5 s/line were measured for a scan area of (5 × 100) µm^2^, resulting in a resolution of 5,120 × 256 points. For each fibre, measurement data were taken from three different locations (at least 2 mm apart from each other). AFM images were initially processed using the open-source software Gwyddion 2.45. A two-dimensional levelling of the data was carried out by mean plane subtraction, and polynomial background subtraction (4th degree horizontal, 6th degree vertical) to remove the macroscopic fibre shape. Afterwards, MATLAB was used for further calculations to obtain the parameters of the scattering model as used in the following.

### Modelling of bent fibres including core-cladding interface roughness

The modelling of light propagation in polymer optical fibres, including scattering effects for arbitrary wavelengths, is a complicated task in the field of wave optics. However, since the diameters of the used fibres are much larger than the wavelength of the transmitted light, ray-tracing can be used to calculate the effect of bending in order to compare it to measurement results. For this purpose, the fibre was approximated by a flexible CAD model, which included the relevant material properties, such as refractive indices and absorption coefficients of all components (see Table 1). With the model in place, a light source (λ = 652 nm) radiating isotropically into all directions was placed at the central axis of the POF immediately behind the front face of the fibre. We kept the incoupling angle < total internal reflection angle, so that no light would reach the cladding before the first bending (see Fig. 4b). For implementation, the commercial software FRED was used (Photon Engineering, LCC: Optical Engineering Software FRED. Version: 2014). It allows the calculation of the attenuation for arbitrary geometries of an optical fibre in dependence of the light source parameters such as wavelength and angular intensity distributions. This ray-tracing software uses the laws of reflection and refraction and includes the Fresnel equations to determine the ray direction and power. Combining these with the consideration of special wave-optics phenomena and statistical scatter models leads to an adequate description of light propagation through the fibre.

For the ray-tracing simulation of light transmission through the fibre, a single ray leaving the light source at a randomly chosen angle was generated and its path through the fibre was registered, see Fig. 4a. When an interface was hit, the ray normally split into two: the reflected and the refracted light. In order to avoid this doubling of the number of rays (and over time, its exponential increase), a Monte-Carlo procedure was applied in order to choose one of the rays for further tracking, whereas the other one was neglected. With this method, the propagation of the ray through the fibre was logged. Finally, at the end of the fibre, both intensity and position of the outgoing ray were registered.

In an ideal POF made from homogeneous material and having ideally smooth interfaces, the propagation of light is described by straight light rays and changes in direction are caused only by refraction and reflection at the interface between different materials according to Snellius’ law. In reality, both inhomogeneities in the materials and the roughness of interfaces cause deviations from this behaviour resulting in additional transmission losses.

Of particular importance is the roughness of the interfaces between core and cladding and/or between cladding and surrounding material, e.g. air. It causes the incoming light to not behave according to the classical laws of refraction and reflection, but to spread around the directions given by Snellius’ law. This stray light is included in the simulation by a statistical scatter model accounting for the direction-dependent intensity of the scattered light. One can show that the specific form of the stray light distribution is determined by the relation between the wavelength of the impinging light and the mean roughness of the material interface, which—for the polymer optical fibre material used here—is of the order of one-tenth or smaller^19,20,22^.

Inhomogeneities in the materials lead to scattering of the light inside the material (“volume scattering”). However, for the highly transparent core material used in the present investigation, these turn out to be negligible, compared to the transmission losses caused by the scattering at the core-cladding- and the cladding-air-interface, respectively (see the corresponding absorption coefficient in Table 1 and references^13,20,23^. In the following, we, therefore, do not include volume scattering in the core into our discussion. On the other hand, volume scattering in the cladding is included and modelled by material absorption (see the much higher absorption coefficient in Table 1).

Repeating this procedure for a large number of emitted rays, the isotropic radiation of the source could be imitated, and by averaging over the rays reaching the end of the fibre, the damping behaviour could be obtained with the required accuracy. We used 10^5^ initial rays for each simulation, leading to a relative uncertainty of computed losses of less than 1%.

The software package FRED used for simulation allows the inclusion of statistical stray light models for the scattering at the material interfaces. For this purpose, the 2-dimensional height profile *h*(*x,y*) of a surface area *A* has to be analyzed.

Given this profile, a number of parameters can be derived, in particular:

- The mean vertical fluctuation, expressed by the rms roughness *S*_*q*_,
  5 $$S_{q}^{2} = \frac{1}{A}\mathop {\iint }\limits_{A}^{{}} h^{2} \left( {x,y} \right)dxdy$$
- The mean lateral size of structures, caused in particular by lateral ripples of the fibre, given by the autocorrelation length *S*_*al*_, i.e. the distance, at which the autocorrelation function *S*_*ACF*_ of the height profile,
  6 $$S_{ACF}^{{}} \left( {t_{x} ,t_{y} } \right) = \frac{1}{A}\mathop {\iint }\limits_{A}^{{}} h\left( {x,y} \right)h\left( {x - t_{x} ,y - t_{y} } \right)dxdy$$
  drops down to a certain value, typically 1/e. Here, *t*_*x*_ and *t*_*y*_ are the spatial shifts in x-and y-direction, respectively.
- The mean slope *sl* of the surface,
  7 $$sl = \frac{{S_{q} }}{{S_{al} }}$$

If the surface is isotropic and the roughness is sufficiently small (< 1/10 of the wavelength λ), the stray light distribution is given by the Bidirectional Scattering Distribution Function (BSDF). It correlates the intensity of the reflected resp. the refracted light for a given direction to the intensity of the 1-dimensional power spectral density (PSD), i.e. the Fourier transform of the height profile along with a scan of length *L*,

8 $$PSD\left( f \right) = \frac{1}{L}\left| {\mathop \smallint \limits_{0}^{L} e^{ - 2\pi ifx} h\left( x \right)dx} \right|^{2} , - \infty < f < \infty$$

at a certain spatial frequency *f* (for details see^20,22^).

For fitting the usually strongly fluctuating PSD the so-called ABC-fit has been established^22^:

9 $$PSD\left( f \right) = \frac{A}{{2\left[ {1 + \left( {Bf} \right)^{2} } \right]^{C/2} }}$$

where the parameters *A* and *B* are related to the roughness parameters *S*_*q*_ and *sl* defined above as follows:

10 $$A = \frac{{4S_{q}^{3} }}{sl}$$

11 $$B = \frac{{2S_{q} \sqrt \pi }}{sl}\frac{{\Gamma \left( {\frac{C - 1}{2}} \right)}}{{\Gamma \left( \frac{C}{2} \right)}}$$

Here, Γ denotes Euler’s Gammafunction whereas *C* is a free fitting parameter of the order of 2, characterizing the decay of the PSD at high frequencies.

In a real fibre, the roughness parameters can be directly measured only for the cladding-air interface. As described above, this measurement was conducted by AFM. The core-cladding interface is inaccessible to direct observation due to the difficulties in separating both materials. Although mechanical, chemical, or laser-induced removal of the cladding is possible, this would alter the interface or leave residues which are in the same order of magnitude as the original roughness itself. Additionally, the spinning of only the core leads to a different thermal gradient along the radius of the fibre^5^ and, consequently, to a different interface. Therefore, we simulated the core-cladding interface with rms roughness (*S*_*q*_) in a range between 0 and 25 nm, as observed for the cladding-air interface, and compared the simulation results to the experimental findings. For this comparison, it is sufficient to consider a straight fibre since the roughness is not changed by bending (or, if changed, the changes may be neglected) and the core-cladding interface roughness is a crucial damping mechanism for the guiding of light in straight fibres.

## Supplementary information

Supplementary Information.

## Data Availability

The datasets generated during and/or analysed during the current study are available from the corresponding authors on reasonable request.
