# Supplementary material for: Experimental determination and ray-tracing simulation of bending losses in melt-spun polymer optical fibres
Source: Sci Rep. 2020 Jul 17;10:11885. doi: 10.1038/s41598-020-68568-0 (PMC7367840; doi:10.1038/s41598-020-68568-0)
Supplement: Supplementary file 1 — Supplementary Information. [file 41598_2020_68568_MOESM1_ESM.docx]

Experimental determination and ray-tracing simulation of bending losses in melt-spun polymer optical fibers

Birgit Lustermann^1,^*^,+^, B. Maike Quandt^2,3,+^, Sebastian Ulrich^2^, Fabrizio Spano^2^, René M. Rossi^2^, and Luciano F. Boesel^2,^*

^1^University of Applied Sciences Nordhausen, Weinberghof 4, 99734 Nordhausen, Germany

^2^Empa, Swiss Federal Laboratories for Materials Science and Technology, Laboratory for Biomimetic Membranes and Textiles, Lerchenfeldstr. 5, 9014 St. Gallen, Switzerland

^3^Empa, Swiss Federal Laboratories for Materials Science and Technology, Laboratory of Advanced Fibers, Lerchenfeldstr. 5, 9014 St. Gallen, Switzerland

[*Birgit.Lustermann@hs-nordhausen.de](mailto:*Birgit.Lustermann@hs-nordhausen.de), [luciano.boesel@empa.ch](mailto:luciano.boesel@empa.ch)

^+^ BL and BMQ contributed equally to this work.

1. Supporting Information

**Table S1. Damping coefficient (in db/cm) ± S (standard error of the linear regression, in db/cm) of regression lines for all curves of Figure 1. Regression equation was *D* = a·*L* (*D* = attenuation in db, *L* = length in cm).**

| Fiber ID | Wire diameter, d_w_ [mm] | | | | |
| --- | --- | --- | --- | --- | --- |
|  |  | | | | |
|  | 0.12 | 0.30 | 0.80 | 1.20 | 1.50 |
| 1143 | 16.8 ± 0.3 | 7.07 ± 0.19 | 2.80 ± 0.05 | 1.68 ± 0.05 | 0.683 ± 0.025 |
| 1144 | 12.8 ± 0.3 | 7.20 ± 0.11 | 1.98 ± 0.04 | 1.04 ± 0.02 | 0.412 ± 0.016 |
|  | *** | *p* = 0.21 | *** | *** | *** |

*** *p* < 0.001

For comparison of the performance of the two fibers, the experimental data in Figure 1 was fitted by linear regression. To test for significance, ANOVA was performed on attenuation as a function of (length)*(fiber ID). The interaction factor (length):(fiber ID) was analysed to indicate significant differences in the damping coefficient of the two fibers when wound around wires with the same diameter. These results are summarized in Table S1. It is important to stress that a linear fit can only provide a very rough approximation to the real behaviour, since the attenuation shows a non-trivial, non-linear dependence both on fiber length and on bending diameter. In section 2.4 we compare the experimental data to the results of our ray-tracing and scattering model based simulations, which allow to discuss the influence of different attenuation mechanisms in much more detail (see Figure 5). The linear fit was used here for comparison purposes only.

**Table S2: Fitting parameters for fiber 1143, together with S (the standard error of the regression of experimental data) for each bending diameter.**

| d_w_ [mm] | P_1_ | p_1_ | P_2_ | p_2_ | P_3_ | p_3_ | S [%] |
| --- | --- | --- | --- | --- | --- | --- | --- |
| 0.12 | 0.496 | 3.06 | 0.382 | 0.511 | 0.122 | 0.0670 | 1.75 |
| 0.30 | 0.246 | 1.48 | 0.575 | 0.289 | 0.179 | 0.0399 | 0.795 |
| 0.80 | 0.0983 | 0.260 | 0.587 | 0.119 | 0.315 | 0.0141 | 1.21 |
| 1.20 | 0.0803 | 0.269 | 0.477 | 0.0756 | 0.442 | 0.00918 | 2.37 |
| 1.50 | 0.0961 | 0.233 | 0.370 | 0.0587 | 0.534 | 0.00733 | 1.53 |

**Table S3: Fitting parameters for fiber 1144, together with S (the standard error of the regression of experimental data) for each bending diameter.**

| d_w_ [mm] | P_1_ | p_1_ | P_2_ | p_2_ | P_3_ | p_3_ | S [%] |
| --- | --- | --- | --- | --- | --- | --- | --- |
| 0.12 | 0.461 | 3.16 | 0.242 | 0.444 | 0.297 | 0.0936 | 1.39 |
| 0.30 | 0.197 | 1.29 | 0.388 | 0.218 | 0.414 | 0.0537 | 4.52 |
| 0.80 | 0.00711 | 0.0801 | 0.601 | 0.0756 | 0.392 | 0.0121 | 2.90 |
| 1.20 | 0.0263 | 0.163 | 0.424 | 0.0570 | 0.550 | 0.00856 | 1.69 |
| 1.50 | 0.0419 | 0.147 | 0.312 | 0.0517 | 0.646 | 0.00693 | 2.05 |


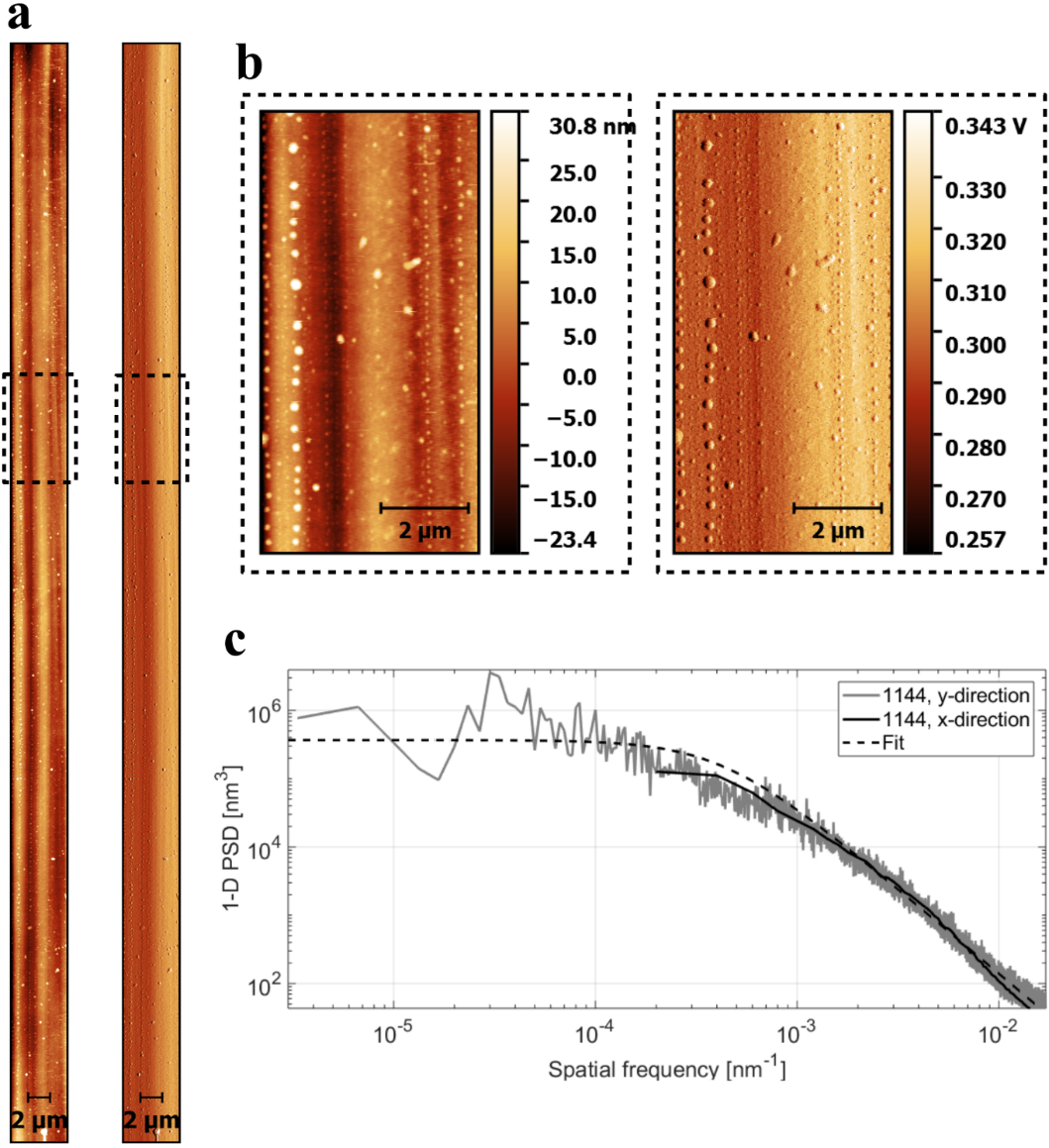


Fig. S1: Exemplary atomic force microscopy (AFM) analysis of Fiber 1144 at one location. (a) Full range AFM image (left: height, right: amplitude). (b) Zoom-in of the height and amplitude AFM images in the indicated (5 x 10) µm^2^ section. The depicted height mode AFM images were further treated by a median of differences row alignment. (c) PSD along x- and y-directions for fiber 1144, together with the corresponding ABC-fit curves.

**
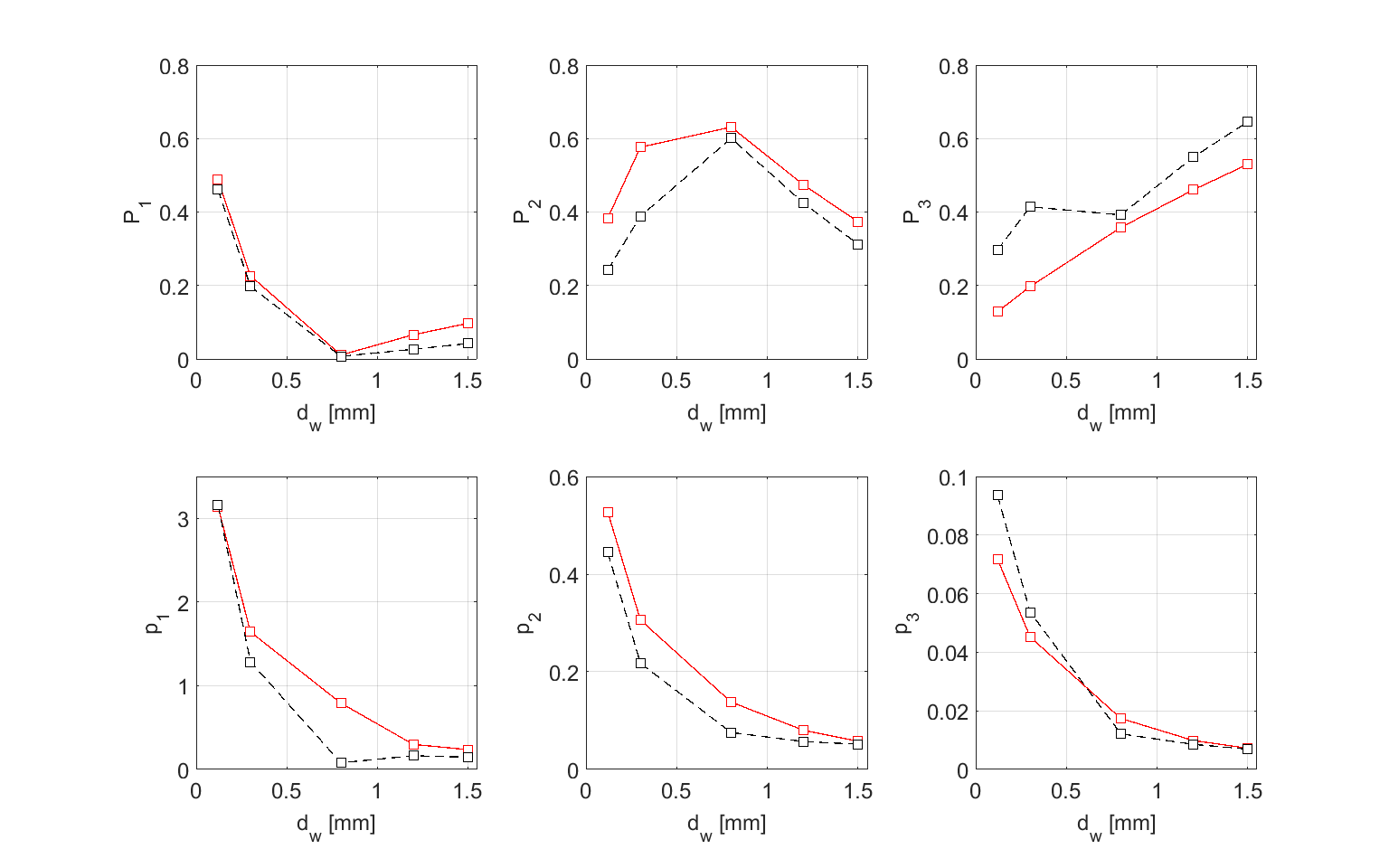
**

Fig. S2: Amplitudes $P_{1}$*,* $P_{2}$*,* $P_{3}$ (top) and exponents *p_1_, p_2_, p_3_* (bottom) of Equation (4) in dependence on the diameter of the bending cylinder *d*_w_. (fibre 1143 – red; fibre 1144 – black-dashed).


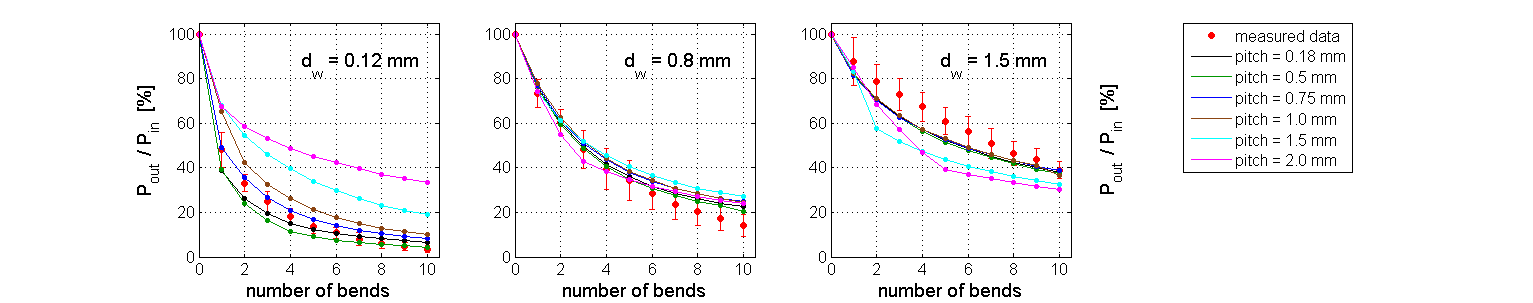


Fig. S3: Exemplary comparison of the experimental bend losses of fibre 1143 for several wire diameters *d_w_* with models incorporating different pitch distances. Red circles: experimental data, mean with s.d., *n* = 10.
